# Downregulation of long non-coding RNA H19 promotes P19CL6 cells proliferation and inhibits apoptosis during late-stage cardiac differentiation via miR-19b-modulated Sox6

**DOI:** 10.1186/s13578-016-0123-5

**Published:** 2016-11-22

**Authors:** Yu Han, Hongdang Xu, Jiangtao Cheng, Yanwei Zhang, Chuanyu Gao, Taibing Fan, Bangtian Peng, Bin Li, Lin Liu, Zhaoyun Cheng

**Affiliations:** 1 Children’s Heart Center, Henan Province People’s Hospital, Zhengzhou, 450003 China; 2 Department of Cardiology, Henan Province People’s Hospital, No. 7 Weiwu Road, Zhengzhou, 450003 China; 3 Department of Ultrasonography, Henan Province People’s Hospital, Zhengzhou, 450003 China; 4 Department of Cardiovascular Surgery, Henan Province People’s Hospital, Zhengzhou, 450003 China

**Keywords:** IncRNA, H19, Cardiac differentiation, miR-19b, Sox6

## Abstract

**Background:**

Regulating cardiac differentiation to maintain normal heart development and function is very important. At present, biological functions of H19 in cardiac differentiation is not completely clear.

**Methods:**

To explore the functional effect of H19 during cardiac differentiation. Expression levels of early cardiac-specific markers Nkx-2.5 and GATA4, cardiac contractile protein genes α-MHC and MLC-2v were determined by qRT-PCR and western lot. The levels of lncRNA H19 and miR-19b were detected by qRT-PCR. We further predicted the binding sequence of H19 and miR-19b by online softwares starBase v2.0 and TargetScan. The biological functions of H19 and Sox6 were evaluated by CCK-8 kit, cell cycle and apoptosis assay and caspase-3 activity.

**Results:**

The expression levels of α-MHC, MLC-2v and H19 were upregulated, and miR-19b was downregulated significantly in mouse P19CL6 cells at the late stage of cardiac differentiation. Biological function analysis showed that knockdown of H19 promoted cell proliferation and inhibits cell apoptosis. H19 suppressed miR-19b expression and miR-19b targeted Sox6, which inhibited cell proliferation and promoted apoptosis in P19CL6 cells during late-stage cardiac differentiation. Importantly, Sox6 overexpression could reverse the positive effects of H19 knockdown on P19CL6 cells.

**Conclusion:**

Downregulation of H19 promoted cell proliferation and inhibited cell apoptosis during late-stage cardiac differentiation by regulating the negative role of miR-19b in Sox6 expression, which suggested that the manipulation of H19 expression could serve as a potential strategy for heart disease.

## Background

The heart comes into being by undergoing many developmental events such as determination of the cardiac field in the mesoderm, differentiation of cardiac precursor cells into cardiomyocytes, and morphogenesis of the chambered heart [1]. Moreover, a series of gene expressions take part in the formation of the mature vertebrate heart with separated chambers and valves [2]. Large numbers of genes play key roles in cardiac morphogenesis, though their precise function and their interaction with other cardiac regulators are not well known [3]. Many embryonic researches have indicated the mechanisms of how and where these steps occur during embryonic development period [4]. However, there is little understanding of the molecular mechanisms that modulate these induced events in the process of the heart formation. P19CL6, a euploid and multipotent mouse cell line, are derived from P19 embryonal carcinoma cells, and DMSO treatment makes P19CL6 differentiate efficiently into cardiac myocytes with obviously spontaneous heartbeat within 10 days [5]. P19CL6 is considered to be a helpful model in vitro to investigate cardiomyocyte differentiation [6].

LncRNAs are a type of approximately 200-nucleotide (nt)-long RNA molecules without protein-coding potential and stably exist in the plasma and urine, and have the characteristics of disease and tissue specificity [7]. Growing research has indicated that lncRNAs are often abnormally expressed in many cancers and play a variety of biological functions in cell proliferation, apoptosis, or invasion [7, 8]. LncRNAs modulate gene expression at multiple levels such as epigenetic, transcriptional and post transcriptional regulation [9]. H19, the first imprinted lncRNA to be defined, is conserved across lineages [10]. Gabory et al. demonstrated that H19^−/−^mice presented an overgrowth phenotype, while overexpressing H19 in transgenic mice caused postnatal growth reduction [11]. It is important for understanding the cardiac development to illuminate the mechanisms of gene regulation. However, the precise function of H19 in cardiac differentiation is yet unascertained.

microRNAs (miRNAs) are endogenous regulatory RNAs, having important effects on heart development and pathogenesis [12]. miR-19b is part of the miR-17-92 group which encodes miR-17, miR-18a, miR-19a, miR-19b, miR-20a and miR-92a-1, and promotes the development of heart, lung, blood vessel and immune system [13]. Cardiogenesis is seriously inhibited in mice lacking miR-17-92, indicating its vital role in cardiac development [13]. A previous study showed that miR-19b overexpression promoted cell proliferation and suppressed apoptosis of mouse embryonic carcinoma cells (P19 cells) [14]. Thus, miR-19b may functionally be connected with cardiogenic processes, although its exact role remains poorly understood.

In this study, we detected the expression levels of Nkx-2.5, GATA4, α-MHC, MLC-2v and H19 in P19CL6 cells at indicated time points. Then we defined the biological effect of H19 in regulating cell proliferation and apoptosis during cardiac differentiation by modulating miR-19b. Moreover, we examined the link between H19 and Sox6 in regulating cell proliferation and apoptosis during cardiac differentiation.

## Methods

### Cell culture and differentiation

P19CL6 mouse embryonic carcinoma cells were grown in Minimum Essential Medium, Alpha Medium (a-MEM; GIBCO, Carlsbad, CA, USA) containing 10% fetal bovine serum (FBS; GIBCO). 293T cells (ATCC; Manassas, VA, USA) were cultured in Dulbecco’s modified Eagle’s medium (DMEM; GIBCO) containing 10% FBS. The cells were kept at 37 °C in a humidified incubator with 5% CO_2_ atmosphere.

To induce P19CL6 cells to differentiate into cardiomyocytes, P19CL6 cells were seeded at a density of 3.5 × 10^5^ cells/well in a six-well plate with the culture medium containing 1% dimethyl sulfoxide (DMSO; Sigma, St. Louis, MO, USA). The conditioned medium was replaced with 2 ml of fresh medium containing 1% DMSO every 2 days. The days of differentiation were numbered consecutively following the first day of the DMSO treatment (day 0).

### Generation of a stable cell line and transfection

Recombinant lentiviruses harboring sh-NC, shH19, H19, si-NC, si-Sox6 or Sox6 were purchased from the GeneChem Company (Shanghai, China). To construct the stable cell line, P19CL6 cells were transfected with lentiviruses and then puromycin (2 µg/ml) was used to select the stable cell line for a week. miR-NC, miR-19b mimics, miR-NC and anti-miR-19b were chemically synthesized by Genechem Company. Briefly, each oligonucleotide was transiently transfected into P19CL6 cells by means of Lipofectamine 2000 (Invitrogen, Carlsbad, CA, USA) at 20 nM according to the manufacturer’s instructions. Samples were collected for qRT-PCR, western blot, cell viability assay or flow cytometry at the indicated time points.

### RNA isolation and quantitative real-time PCR

For mRNA analysis, total RNA was extracted from P19CL6 cells using TRIzol reagent (Thermo Fisher Scientific, Waltham, MA, USA). The reverse-transcription reactions were carried out using a TaqMan™ microRNA assay kit (Applied Biosystems, Foster City, CA, USA) and a Prime Script™ RT reagent kit (Takara, Shiga, Japan) according to the instructions from the respective manufacturers. Quantitative PCR was carried out in a final volume of 20 μl containing 1 μl cDNA, 0.5 μl primers (2.5 μM) and 10 μl SYBR premix exTaq (TaKaRa, Dalian, China) following the manufacturer’s instructions.

### CCK‑8 assay

Cell viability was detected using Cell Counting kit-8 (CCK-8; Dojindo, Kumamoto, Japan) following the manufacturer’s instructions. P19CL6 cells transfected with H19, shH19, Sox6 or si-Sox6 were plated in 96-well plates and maintained in α-MEM containing 10% FBS, 100 U/ml penicillin and 100 μg/ml streptomycin for 4, 6, 8, and 10 d. Briefly, the CCK-8 solution (10% of the medium, 10 µl) was added to each well and incubated for 4 h prior to analysis. Then the absorbance at 450 nm was measured.

### Flow cytometry analysis of apoptosis and cell cycle

For apoptosis analysis, cultured cells were trypsinized and washed with phosphate-buffered saline (PBS). Cells from each sample were processed with the Annexin V-FITC/PI apoptosis detection kit (BD Biosciences, San Jose, CA, USA) as the manufacturer’s instructions at indicated times.

For cell cycle analysis, collected cells were washed twice with PBS and fixed in 75% ethanol at −20 °C overnight. Then cells were harvested and resuspended in 500 µl PBS containing 0.1% RNase (Sigma, St. Louis, MO, USA) for RNA digestion. Finally, cells were stained with 500 µl propidium iodide (PI) solution (50 µg/ml, Sigma) for 15 min at room temperature in the dark. Cell cycle distribution of the cells was then analyzed using FACS Calibur (BD Biosciences) at indicated times.

### Caspase-3 assay

Caspase-3 activity in P19CL6 cells was analyzed by means of Caspase-3 Colorimetric Assay kit (KeyGen, Nanjing, China) following the manufacturer’s instructions. In brief, cells were lysed in ice bath for 1 h and vortexed every 20 min. Then the tissue lysates were centrifuged for 10 min at 12,000*g* at 4 °C. The supernatant were diluted to 50 µl using cell lysis buffer, incubated with 5 µl of substrate at 37 °C for 4 h in dark and a microplate reader (DNM-9602; Beijing Perlong Medical Instrument Ltd, Beijing, China) was used to determine the absorbance of the samples at 405 nm to quantify the caspase-3 activity.

### Luciferase assay

The wild-type H19-3′UTR (WT), mutant H19-3′UTR (MUT), wild-type Sox6-3′UTR (WT) and mutant Sox6-3′UTR (MUT) containing the putative binding site of miR-19b were established and cloned in pmirGLO dual luciferase miRNA reporter vectors (Promega, Madison, WI, USA). The reporter vectors and miR-19b mimics or miR-NC were co-transfected into 293T cells using Lipofectamine 2000 (Invitrogen). After 36 h of incubation, cells were collected and lysed for luciferase activity detection (Promega).

### Antibodies and western blot analysis

Total protein was extracted from cells and protein concentration was analyzed by a NanoDrop 2000 spectrophotometer (Thermo Scientific, Delaware, USA). For western blot analysis, 50 µg of proteins were separated and transferred onto polyvinylidene difluoride membrane (PVDF; Millipore, Billerica, MA, USA). Following blocking for 1 h in PBS with 0.1% Tween 20 (PBST) and 5% BSA, the membranes containing proteins of interest were incubated overnight with specified primary antibody at 4 °C. PVDF membranes were washed in TBST and incubated with secondary antibodies labeled with HRP and detected by ECL (Pierce, Rockford, IL, USA). Antibodies used in this study are Nkx-2.5 (1:500; Santa Cruz Biotechnology, Santa Cruz, CA, USA), GATA4 (1:500; Santa Cruz Biotechnology), α-MHC (1:500; Santa Cruz Biotechnology), MLC-2v (1:500; Santa Cruz Biotechnology), Sox6 (1:10,000; CST, Inc., Danvers, MA, USA), and β-actin (1:10,000; CST, Inc.).

### Statistical analysis

Data were presented as mean ± SD of at least three experiments. The differences between different groups were analyzed using student’s t test and analysis of variance (ANOVA). P < 0.05 was considered statistically significant.

## Results

### H19 was highly expressed in the late stage of cardiac differentiation in P19CL6 cells

**Fig. 1:**
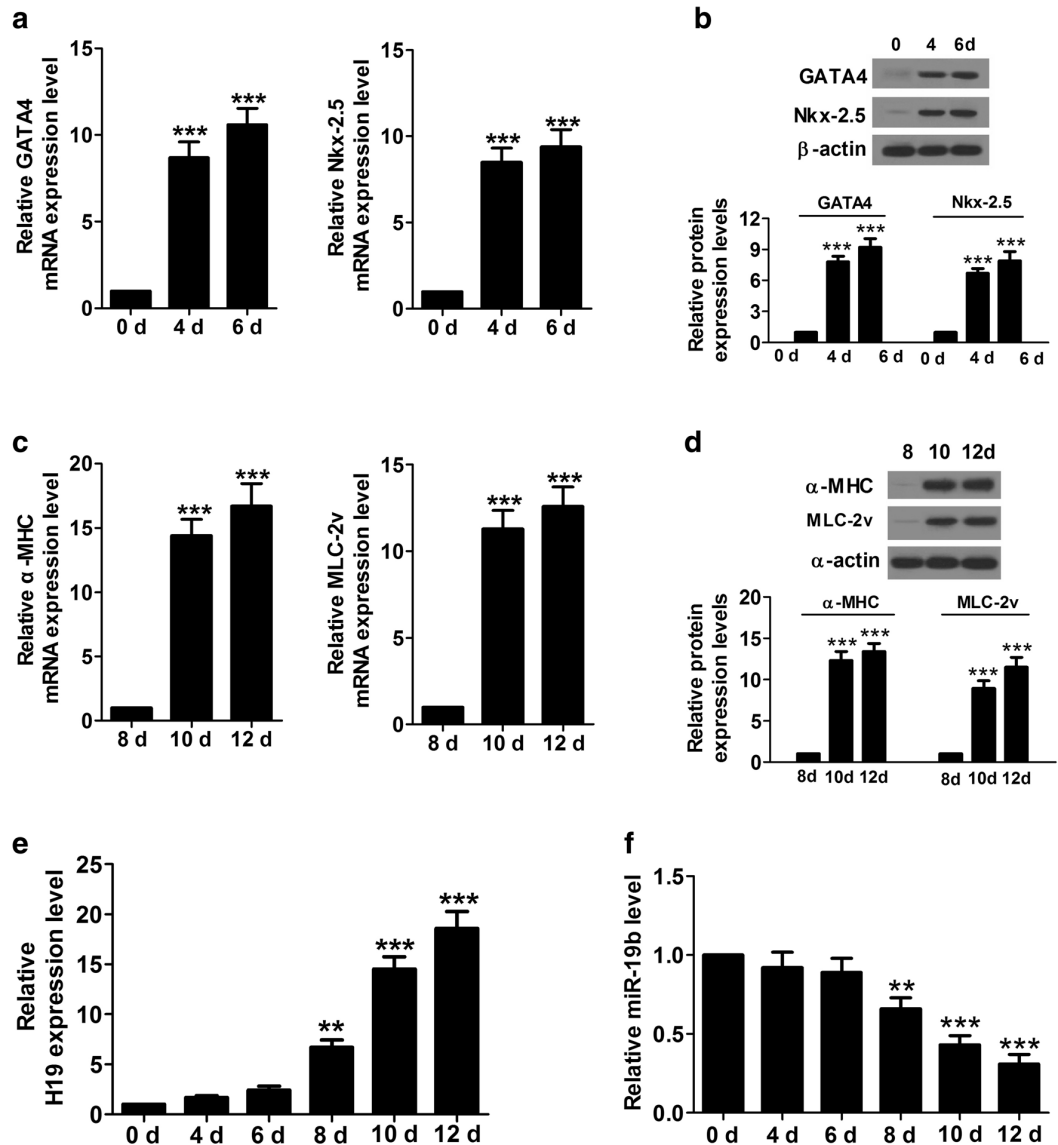
The expression levels of GATA4, Nkx-2.5, α-MHC, MLC-2v and H19 are significantly upregulated and miR-19b was downregulated at indicated time points. **a** mRNA levels of early cardiac-specific markers (GATA4 and Nkx-2.5α-MHC) were increased significantly in P19CL6 cells at day 4 and 6. **b** Protein levels of GATA4 and Nkx-2.5α-MHC were augmented significantly in P19CL6 cells at day 4 and 6. **c** mRNA levels of cardiac contractile protein genes (α-MHC and MLC-2v) were increased significantly in P19CL6 cells at day 10 and 12. **d** Protein levels of α-MHC and MLC-2v were elevated significantly in P19CL6 cells at day 10 and 12. **e** Level of H19 was augmented significantly from day 8 to day 12.** f** Level of miR-19b was dropped significantly from day 8 to day 12. **P < 0.01, ***P < 0.001

We treated P19CL6 cells with 1% dimethyl sulfoxide (DMSO) to induce differentiation. qRT-PCR was carried out to detect the expression levels of early cardiac-specific markers (GATA4 and Nkx-2.5), cardiac contractile protein genes (α-MHC and MLC-2v) in P19CL6 cells at indicated time points. The results showed that mRNA and protein levels of GATA4 and Nkx2.5 were very low at day 0, but increased significantly at day 4 and day 6, an early stage of differentiation (Fig. 1a and b). mRNA and protein levels of α-MHC and MLC-2v in P19CL6 cells were significantly higher at day 10 and 12 (an late stage of differentiation) than those in P19CL6 cells at day 8 (Fig. 1c and d). In order to define the temporal expression profile of H19 during cardiomyocyte differentiation, we carried out qRT-PCR for H19. The expression of H19 was low at day 0, 4 and 6, but elevated significantly from day 8 to day 12 (Fig. 1e). Moreover, the level of miR-19b was significantly reduced from day 8 to day 12, suggesting that H19 and miR-19b might play some biological roles during the late stage of cardiac differentiation of P19CL6 cells (Fig. 1f).

### Knockdown of H19 promoted P19CL6 cell proliferation and inhibits apoptosis

**Fig. 2:**
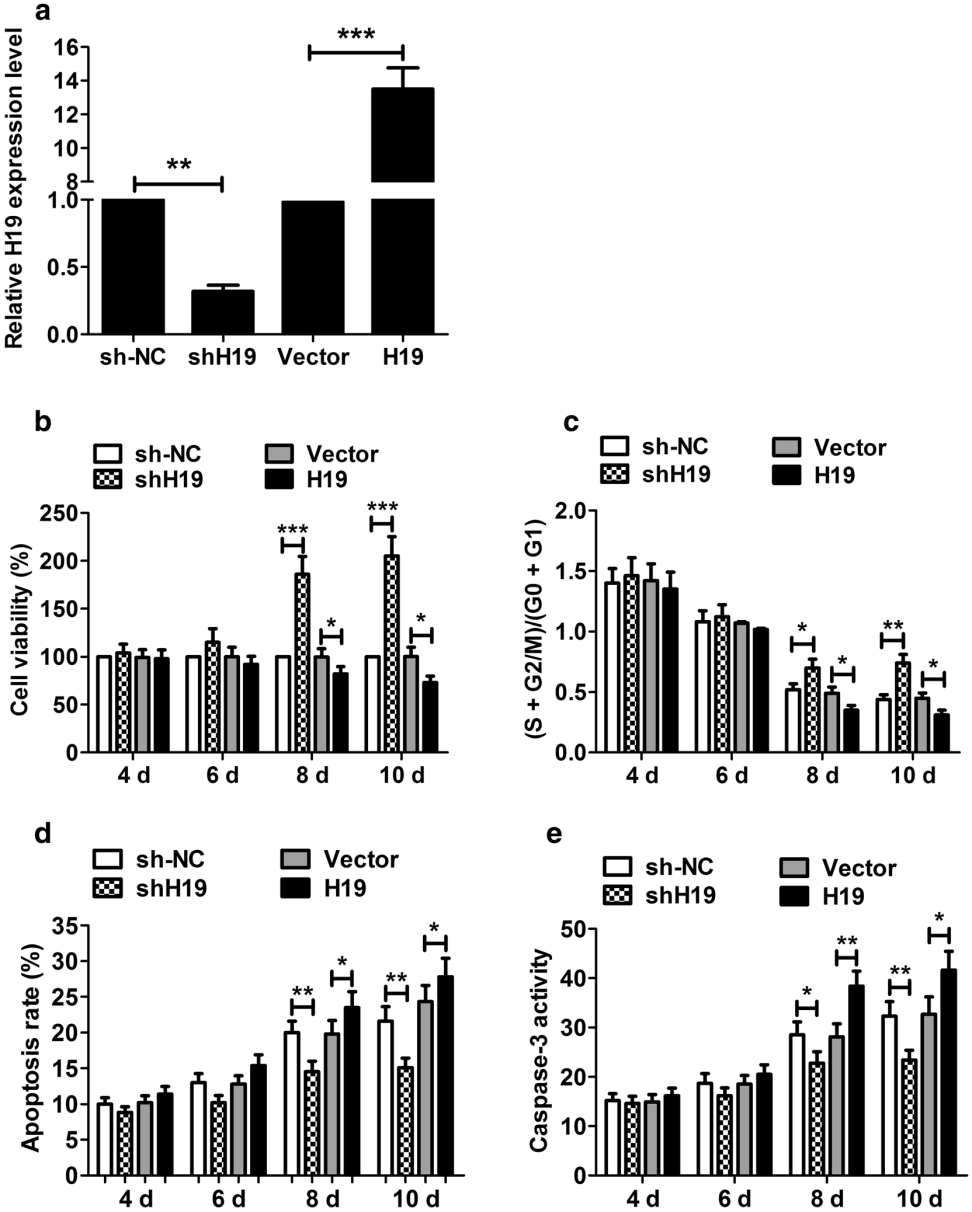
The effect of H19 knockdown and H19 overexpression on P19CL6 cell proliferation and apoptosis. **a** Transfecting P19CL6 cells with shH19 and pcDNA-H19 efficiently down- and up-regulated the H19 level at day 10, respectively. **b** CCK-8 assay showed that shH19 resulted in an increase in cell viability at day 8 and 10, whereas pcDNA-H19 had an opposite effect. **c** Flow cytometry suggested that shH19 significantly increased the cell number in S phase at day 8 and 10, and pcDNA-H19 significantly decreased the cell number. **d** shH19 significantly reduced the apoptotic rate of P19CL6 cells at day 8 and day 10, whereas pcDNA-H19 had an opposite effect. **e** Caspase-3 assay indicated that the activity of caspase-3 was significantly reduced in P19CL6 cells transfected with shH19 and significantly increased in cells transfected with pcDNA-H19. *P < 0.05, **P < 0.01, ***P < 0.001

Given that H19 might participate in the cardiac differentiation of P19CL6 cells, P19CL6 cells were transfected with the shH19 or pcDNA-H19. qRT-PCR results showed that H19 expression was significantly decreased in P19CL6 cells transfected with shH19 and H19 expression was significantly increased in P19CL6 cells transfected with pcDNA-H19 (Fig. 2a).

CCK-8 assay showed that cell viability was significantly enhanced in P19CL6 cells with shH19 and significantly reduced in P19CL6 cells with pcDNA-H19 at day 8 and 10, with no significantly difference at day 4 and 6 (Fig. 2b). Flow cytometry showed that the percentage of (S + G2/M)/(G0 + G1) was significantly increased in P19CL6 cells with shH19 and significantly decreased in P19CL6 cells with pcDNA-H19 at day 8 and 10, with no significantly difference at day 4 and 6 (Fig. 2c). These results suggested that that H19 overexpression significantly inhibited cell proliferation and H19 knockdown significantly promoted cell proliferation in the late stage of differentiation but not in the early stage.

Cell apoptosis assay showed that shH19 significantly reduced the apoptotic rate of cells (Fig. 2d) and the caspase-3 activity (Fig. 2e) at day 8 and day 10, and the opposite effect was observed in pcDNA-H19 cells. Therefore, these results indicated that H19 overexpression significantly promoted cell apoptosis and H19 knockdown significantly inhibited cell apoptosis during the late stage of differentiation but not in the early stage.

### H19 inhibited miR-19b expression

**Fig. 3:**
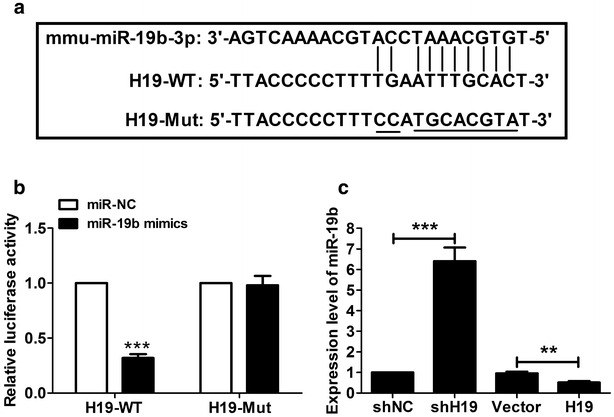
H19 targets and inhibits miR-19b expression. **a** The sketch map of the mmu-miR-19b-3p site in H19 3′-UTR. **b** Luciferase activity assay was performed in 293T cells at 48 h after transfection. miR-19b mimics were capable of significantly inhibiting luciferase expression of H19-WT reporter plasmid but not the H19-Mut reporter plasmid.** c** miR-19b levels were significantly up- or downregulated in P19CL6 cells transfected with shH19 or H19, respectively. **P < 0.01, ***P < 0.001

Online software starBase v2.0 predicted H19 contains binding sequences complementary to miR-19b seed regions, as shown in Fig. 3a. Furtherly, the luciferase report assay indicated that miR-19b mimics inhibited luciferase activity of the wild type H19 reporter (H19-WT), but no change was observed for the luciferase activity in the mutant reporter (H19-Mut) (Fig. 3b). Moreover, the expression level of miR-19b was significantly increased in cells transfected with shH19, whereas significantly reduced in cells with pcDNA-H19. These results indicated that H19 negatively modulated miR-19b expression.

### H19 targets miR-19b to regulate the expression of Sox6

**Fig. 4:**
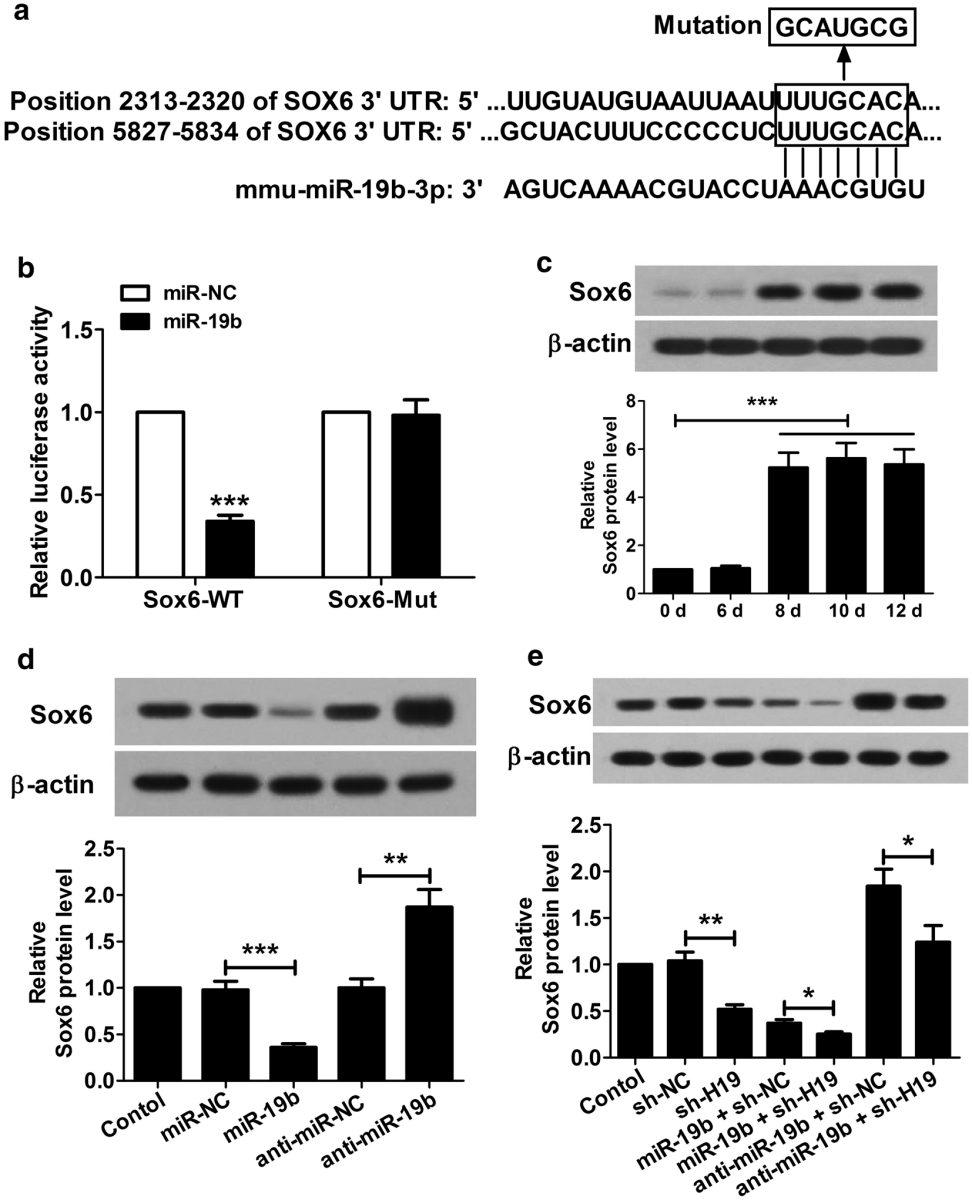
miR-19b targets Sox6 gene and inhibits its expression. **a** The sketch map of the mmu-miR-19b-3p site in Sox6 3′-UTR. **b** Luciferase activity assay was performed in 293T cells at 48 h after transfection. The reporter assay indicated that miR-19b mimics were capable of significantly inhibiting luciferase expression of the Sox6-WT reporter plasmid but not the Sox6-Mut reporter plasmid. **c** Western blot showed that protein level of Sox6 was low from day 0 to 6, and was significantly upregulated from day 8 to 12. **d** The Sox6 protein levels were significantly down- and up-regulated respectively in P19CL6 cells transfected with miR-19b and anti-miR-19b at day 10. **e** shH19 inhibited the Sox6 expression significantly, but miR-19b enhanced the inhibitory effect of shH19 on Sox6 expression and anti-miR-19b reversed the influence of shH19 on Sox6 expression at day 10 after transfection. *P < 0.05, **P < 0.01, ***P < 0.001

Bioinformatics analysis using online software TargetScan indicated that miR-19b may bind to 3′UTR of Sox6 (Fig. 4a). In addition, luciferase reporter assay indicated that miR-19b significantly suppressed luciferase expression of the wild type Sox6 reporter (Sox6-WT) but not the mutant reporter (Sox6-Mut) (Fig. 4b), confirming that Sox6 was a target of miR-19b. Western blot was used to detect the expression of Sox6 in the cell differentiation and the results indicated that the protein level of Sox6 was very low from day 0 to day 6, but increased significantly from day 8 to day 12 (Fig. 4c). Then we transfected P19CL6 cells with miR-19b mimics, miR-NC, anti-miR-19b or anti-miR-NC. The results showed that the protein level of Sox6 was significantly down-regulated in P19CL6 cells transfected with miR-19b and significantly up-regulated in P19CL6 cells transfected with anti-miR-19b (Fig. 4d). Furthermore, we transfected H19-knockdown P19CL6 cells with miR-19b mimics or anti-miR-19b. The result showed that knockdown H19 inhibited the Sox6 expression significantly, while miR-19b enhanced the inhibitory effect of knockdown H19 on Sox6 expression, and anti-miR-19b reversed the influence of knockdown H19 on Sox6 expression (Fig. 4e). All these results suggested that knockdown of H19 inhibited the Sox6 expression by modulating miR-19b.

### Knockdown of Sox6 promoted P19CL6 cell proliferation and inhibits apoptosis

**Fig. 5:**
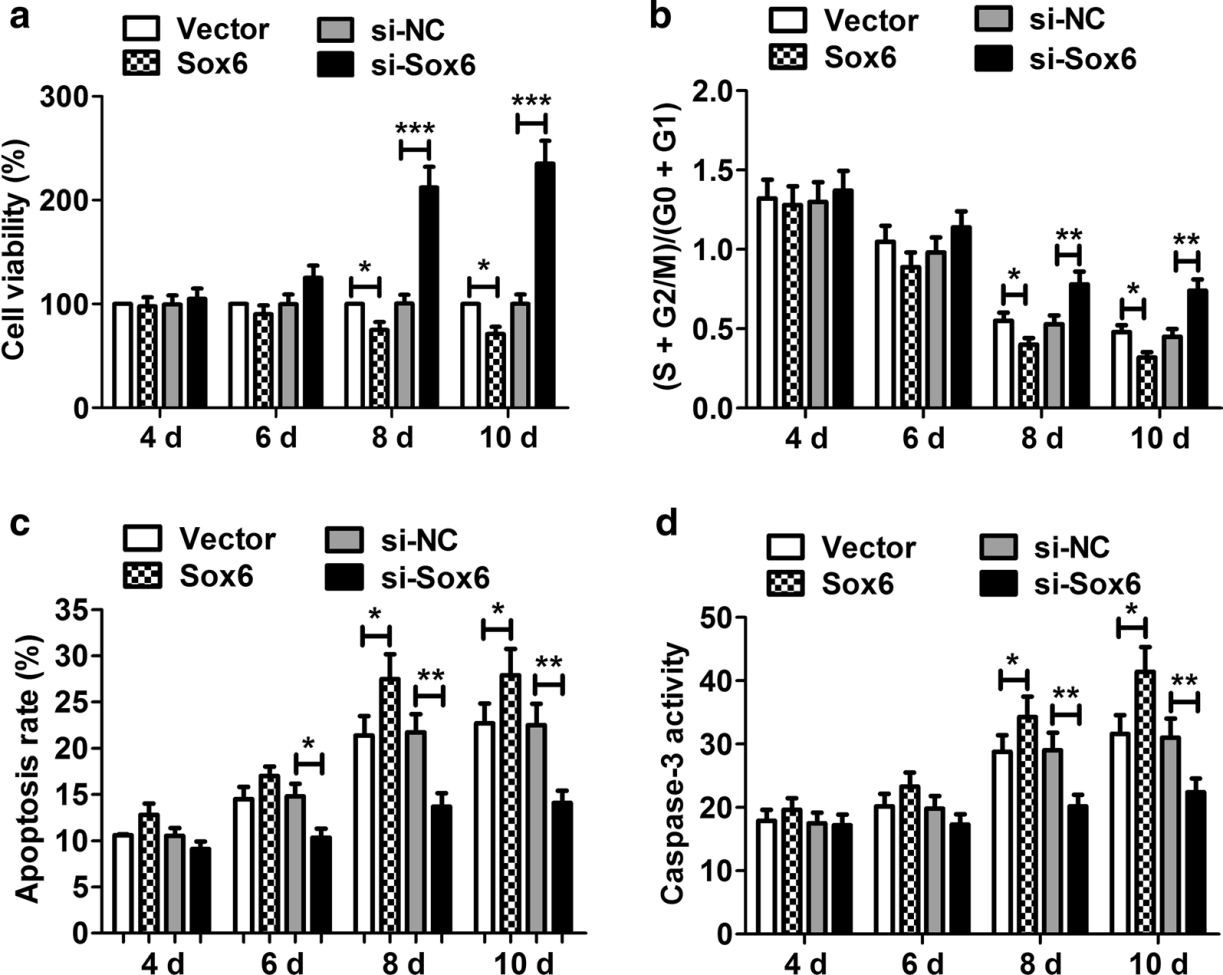
The effect of Sox6 knockdown and overexpression on P19CL6 cell proliferation and apoptosis. **a** CCK-8 assay showed that pcDNA-Sox6 significantly inhibited cell viability at day 8 and 10, and si-Sox6 significantly promoted cell viability at day 8 and 10. **b** Cell cycle analysis found that pcDNA-Sox6 significantly decreased the cell number in S phase at day 8 and 10, and si-Sox6 obviously increased the cell number. **c** Flow cytometry showed that si-Sox6 significantly reduced the percentage of apoptotic cells from day 6 to 10. However, pcDNA-Sox6 played an opposite role in apoptosis at day 8 and 10. **d** Activity of caspase-3 was significantly reduced in cells transfected with si-Sox6 and significantly enhanced in cells transfected with pcDNA-Sox6. *P < 0.05, **P < 0.01, ***P < 0.001

We transfected P19CL6 cells with pcDNA-Sox6 or si-Sox6. CCK-8 viability assay showed pcDNA-Sox6 significantly inhibited cell viability and si-Sox6 significantly promoted cell viability at day 8 and 10 (Fig. 5a). Flow cytometer showed found that pcDNA-Sox6 reduced the number of cells in S phase and si-Sox6 increased the percentage of cells in (S + G2/M)/(G0 + G1) at day 8 and 10 (Fig. 5b). As shown in Fig. 5c and d, si-Sox6 significantly decreased the percentage of apoptotic cells and the activity of caspase-3 at day 8 and 10, while pcDNA-Sox6 significantly enhanced the percentage of apoptotic cells and the activity of caspase-3 compared with the control group. All these results indicated Sox6 knockdown promoted P19CL6 cells proliferation and inhibited apoptosis.

### Sox6 reversed the proliferation and anti-apoptosis effects of of H19 knockdown on P19CL6 cell

**Fig. 6:**
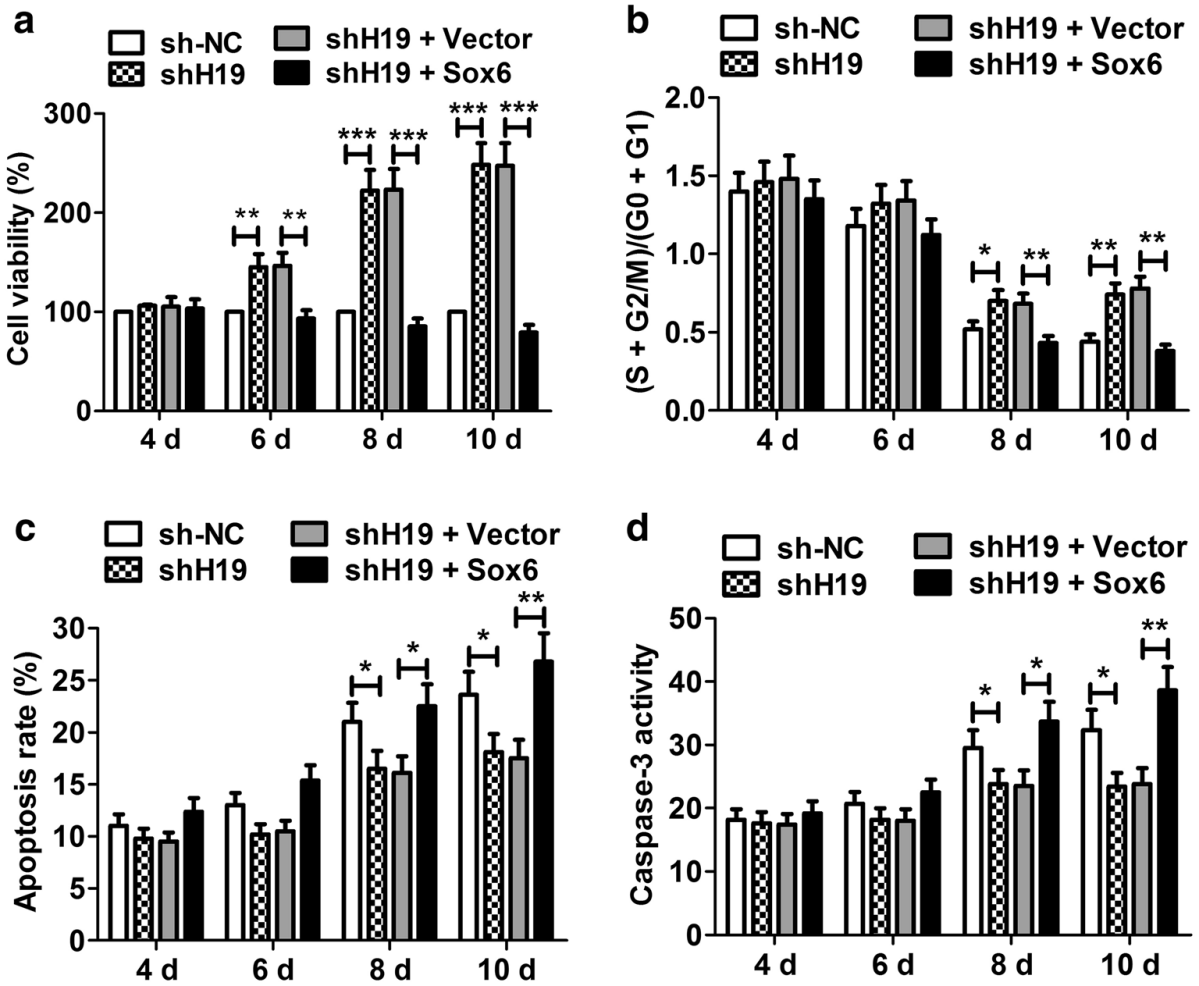
Sox6 reversed the proliferation and antiapoptosis effects of H19 knockdown on P19CL6 cells. **a** CCK-8 assay indicated shH19 boosted the viability of P19CL6 cells at day 8 and day 10, and pcDNA-Sox6 reversed the effect. **b** Cell cycle assay found that shH19 increased the percentage of cells in the S phase at day 8 and 10, whereas pcDNA-Sox6 attenuated the effect. (**c** and **d**) The rate of apoptosis and caspase-3 activity were significantly decreased in cells treated with shH19 at day 8 and 10, and pcDNA-Sox6 overturned the effect. *P < 0.05, **P < 0.01, ***P < 0.001

To further testify the link between H19 and Sox6, we transfected shH19 or shH19 + pcDNA-Sox6 into P19CL6 cells to investigate their effects on cell proliferation and apoptosis. CCK-8 assay indicated that shH19 significantly promoted the viability of P19CL6 cells at day 6, 8 and 10, but pcDNA-Sox6 attenuated the effect of shH19 on cell viability (Fig. 6a). Cell cycle assay indicated that shH19 markedly increased the percentage of cells in (S + G2/M)/(G0 + G1), while pcDNA-Sox6 overturned the effect (Fig. 6b). Futhermore, we found that the rate of apoptosis and caspase-3 activity were obviously reduced in cells treated with shH19, however, pcDNA-Sox6 reversed the anti-apoptosis effect of shH19 on P19CL6 cells (Fig. 6c and d). All of the data suggested that H19 regulated P19CL6 cell proliferation and apoptosis by modulating Sox6 expression.

### Discussion/conclusion

It is necessary for heart development that a regular process essentially exists during cardiac differentiation. Furthermore, cardiac differentiation has been comprehensively investigated. In our study, our aim was to define the connection between H19 and miR-19b and its biological effect on P19CL6 during late-stage cardiomyocyte differentiation. One study showed that H19 had an inhibitory effect on the proliferation of fetal liver cells during liver developments [15]. Similarly, the results presented by Gabory et al. confirmed that H19 played a negative role in cell proliferation [11]. Lately, it was reported that knockdown of H19 accelerated differentiation of parthenogenetic embryonic stem cells (p-ESCs) to cardiomyocytes [16, 17]. Nevertheless, little is known about the precise molecular mechanisms of H19 in cardiomyocyte differentiation. In this study, upregulated expression of H19 in P19CL6 cells was observed at the late stage of differentiation (day 8, 10 and 12). Then, further experiments were performed to detect the roles of knockdown of H19 in cell proliferation and apoptosis existing in cardiomyocyte differentiation. Indeed, knockdown of H19 promoted cell proliferation and inhibited apoptosis at the late stage of differentiation, but not the early stage.

There have been some reports that miRNAs can promote cardiac development and be used as new biomarkers and therapeutic targets for coronary heart disease (CHD) [18]. The results presented by Joost et al. [19] showed that upregulation of miR-1 and -499 inhibited cardiomyocyte progenitor cell (CMPC) proliferation and promoted differentiation into cardiomyocytes in human CMPCs and embryonic stem cells, possibly by inhibition of histone deacetylase 4 or Sox6. CAI et al. found that miR-124 was down-regulated in bone marrow-derived mesenchymal stem cells (BMSCs), targeted STAT3 and in turn influenced the levels of ANP, TNT, a-MHC, and GATA-4 for the first time. [20]. Recently, other investigators showed that miR-10a could target and inhibit effectively GATA6 expression, which reduced the proliferation of hCMPCs during heart development [21]. Therefore, miRNAs play a vital role in heart development, but the specific mechanism is not completely clear. In this study, H19 suppressed the expression of miR-19b. Furthermore, we also confirmed that miR-19b could target directly Sox6 and inhibited its expression.

Sox6, a member of Sox transcription-factor family, is a multi-faceted transcription factor participating in the terminal differentiation of many different cell types in vertebrates [22]. Sox6 without the transactivation or transrepression domain is different from the majority of transcription factors, but interacts with other cofactors to collaboratively regulate target genes [23]. It is reported that Sox6 plays a tumor-suppressive role in cancers [24, 25]. The results presented by Qin et al. showed that Sox6 suppressed cell proliferation by upregulating expression levels of p53 and p21 and down-regulating expression levels of cyclin D1/CDK4, cyclin A, and β-catenin in esophageal cancer [26]. Recently, investigators reported that Sox6 played vital roles in cell differentiation and proliferation [27, 28]. In agreement with this, our result indicated that Sox6 down-regulation significantly decreased the percentage of apoptotic cells and promoted cell proliferation during late stage of cardiomyocyte differentiation, whereas overexpression of Sox6 played the opposite roles. Importantly, Sox6 overexpression reversed the effects of H19 knockdown on P19CL6 growth and apoptosis during late stage of cardiomyocyte differentiation.

In conclusion, we defined that H19 knockdown promoted P19CL6 cells proliferation and inhibited apoptosis by modulating miR-19b. Furthermore, Sox6, as a miR-19b target, could inhibit P19CL6 cells proliferation and promoted apoptosis. Thus, our data indicated that H19 could inhibit miR-19b expression, and miR-19b targets Sox6, regulating cell proliferation and apoptosis during late stage of cardiomyocyte differentiation. This study provides a theoretical basis for the research on lncRNA in the heart development.

## Abbreviations

BMSCs: bone marrow-derived mesenchymal stem cells
CCK-8: cell counting kit-8
FBS: fetal bovine serum
DMEM: Dulbecco’s modified Eagle’s medium
DMSO: dimethyl sulfoxide
PVDF: polyvinylidene difluoride membrane
PBS: phosphate-buffered saline
p-ESCs: parthenogenetic embryonic stem cells
CHD: coronary heart disease
CMPC: cardiomyocyte progenitor cell
